# Dasatinib enhances tumor growth in gemcitabine-resistant orthotopic bladder cancer xenografts

**DOI:** 10.1186/s13104-016-2256-3

**Published:** 2016-09-27

**Authors:** Stefan Vallo, Martin Michaelis, Kilian M. Gust, Peter C. Black, Florian Rothweiler, Hans-Michael Kvasnicka, Roman A. Blaheta, Maximilian P. Brandt, Felix Wezel, Axel Haferkamp, Jindrich Cinatl

**Affiliations:** 1 Institute of Medical Virology, Goethe University Frankfurt, Paul-Ehrlich-Str. 40, 60596 Frankfurt am Main, Germany; 2 Department of Urology, Goethe University Frankfurt, Frankfurt, Germany; 3 Centre for Molecular Processing and School of Biosciences, University of Kent, Canterbury, UK; 4 Department of Urologic Sciences, Vancouver Prostate Centre, University of British Columbia, Vancouver, Canada; 5 Dr. Senckenberg Institute of Pathology, Goethe University Frankfurt, Frankfurt am Main, Germany; 6 Department of Urology, University Hospital Ulm, Ulm, Germany

**Keywords:** Acquired resistance, Cancer cell line collection, Dasatinib, Gemcitabine, Orthotopic xenograft model, Urothelial bladder cancer

## Abstract

**Background:**

Systemic chemotherapy with gemcitabine and cisplatin is standard of care for patients with metastatic urothelial bladder cancer. However, resistance formation is common after initial response. The protein Src is known as a proto-oncogene, which is overexpressed in various human cancers. Since there are controversial reports about the role of Src in bladder cancer, we evaluated the efficacy of the Src kinase inhibitor dasatinib in the urothelial bladder cancer cell line RT112 and its gemcitabine-resistant sub-line RT112^r^GEMCI^20^ in vitro and in vivo.

**Methods:**

RT112 urothelial cancer cells were adapted to growth in the presence of 20 ng/ml gemcitabine (RT112^r^GEMCI^20^) by continuous cultivation at increasing drug concentrations. Cell viability was determined by MTT assay, cell growth kinetics were determined by cell count, protein levels were measured by western blot, and cell migration was evaluated by scratch assays. In vivo tumor growth was tested in a murine orthotopic xenograft model using bioluminescent imaging.

**Results:**

Dasatinib exerted similar effects on Src signaling in RT112 and RT112^r^GEMCI^20^ cells but RT112^r^GEMCI^20^ cells were less sensitive to dasatinib-induced anti-cancer effects (half maximal inhibitory concentration (IC_50_) of dasatinib in RT112 cells: 349.2 ± 67.2 nM; IC_50_ of dasatinib in RT112^r^GEMCI^20^ cells: 1081.1 ± 239.2 nM). Dasatinib inhibited migration of chemo-naive and gemcitabine-resistant cells. Most strikingly, dasatinib treatment reduced RT112 tumor growth and muscle invasion in orthotopic xenografts, while it was associated with increased size and muscle-invasive growth in RT112^r^GEMCI^20^ tumors.

**Conclusion:**

Dasatinib should be considered with care for the treatment of urothelial cancer, in particular for therapy-refractory cases.

## Background

Bladder cancer is the second most common cancer of the genitourinary tract. First-line therapy regimens for metastatic disease include gemcitabine and cisplatin (GC) as standard of care resulting in response rates of 40–70 %. However, resistance acquisition is common and the median survival is unsatisfactory being 12–14 months [1, 2]. Effective second-line therapies are missing [3, 4].

The c-Src proto-oncogene has been strongly implicated in the development, growth, progression, and metastasis of a number of human cancers including those of colon, breast, pancreas, and brain [5, 6]. Several clinical trials showed antitumoral activity of Src family inhibitor dasatinib in different cancer entities [7, 8]. The role of Src kinase as drug target in bladder cancer is controversial. Src inhibition caused anti-cancer effects in some bladder cancer cell lines (including RT112) and animal models [9, 10]. In other studies, however, Src inhibition increased bladder cancer cell migration and metastasis formation [11, 12]. A recently published phase II trial did not demonstrate clinical benefit of single-agent dasatinib in unselected patients with muscle-invasive urothelial carcinoma of the bladder [13].

Here, we investigated the effects of dasatinib on RT112 urothelial bladder cancer cells and RT112 cells with acquired resistance to gemcitabine (RT112^r^GEMCI^20^) in cell culture and in an orthotopic xenograft model.

## Methods

### Drugs

Gemcitabine (Gemzar^®^) was obtained from Lilly (Indianapolis, IN, USA). Dasatinib was obtained from Absource Diagnostics (München, Germany).

### Cell lines and lentiviral transduction

RT112 cells were obtained from the American Type Culture Collection (Manassas, VA, USA). Cells were grown in Iscove’s modified Dulbecco’s medium supplemented with 10 % fetal calf serum (FCS, Gibco, Karlsruhe, Germany). RT112 cells were adapted to growth in presence of 20 ng/ml gemcitabine by continuous cultivation in the presence of increasing drug concentrations as previously described [14] resulting in the gemcitabine-resistant subline RT112^r^GEMCI^20^. For in vivo studies, RT112 and RT112^r^GEMCI^20^ cell lines underwent transduction with a lentiviral construct carrying the luciferase firefly gene for in vivo imaging resulting in cell lines RT112luc and RT112^r^GEMCI^20^luc. The luciferase plasmid contained a blasticidin-resistance gene enabling positive selection with 10 mg/ml blasticidin (Life Technologies GmbH, Darmstadt, Germany). Cell lines were controlled for in vitro luciferase activity and cell number was correlated with bioluminescence using the Xenogen IVIS Spectrum (Caliper Lifesciences, Hopkinton, MA, USA) as previously described [15].

### Viability assay

Cell viability was tested by 3-(4,5-dimethylthiazol-2-yl)-2,5-diphenyltetrazolium bromide (MTT) dye reduction assay after 120 h incubation modified as described before [16]. All experiments were performed at least in triplicate.

### Cell proliferation

At day 0, 4000 cells/ml were plated in culture flasks. Cell numbers were determined every 24 h for 6 consecutive days using the automated cell counter Countess^®^ (Life Technologies GmbH) after trypan blue staining. Doubling time (DT) was calculated using the formula DT = culture time/cell doubling. Cell doubling = ln(Nf/Ni)/ln2, where Ni represents seeded cells number and Nf the harvested cells number [17]. All experiments were performed at least in triplicate.

### Western blot

Cells were lysed in Triton X sample buffer and separated by sodium dodecyl sulfate polyacrylamide gel electrophoresis (SDS-PAGE). Proteins were detected using specific antibodies against β-actin (#A5441, Sigma-Aldrich, St. Louis, MO, USA), Src (#2123, Cell Signaling, Cambridge, UK), phosphorylated Src (Tyr416, #2101, Cell Signaling), Akt (#9272, Cell Signaling), and phosphorylated Akt (Thr308, #2965, Cell Signaling and Ser473, #04-736, EMD-Millipore, Billerica, MA, USA). Protein bands were visualized by enhanced chemiluminescence using a commercially available kit (GE Healthcare, Little Chalfont, UK). Pixel density of western blots is given in percentage compared to untreated cell line (100 %). All experiments were performed at least in triplicate.

### Wound healing migration assay

RT112 or RT112^r^GEMCI^20^ cells were plated onto six-well plates and allowed to form a confluent monolayer. The cell monolayer was then scratched in a straight line to make a ‘scratch wound’ with a 0.2 ml pipette tip and the cell debris was removed by washing the cells with phosphate-buffered saline. Images of the closure of the scratch were captured at 0, 6, and 24 h. Quantification of wound repair was obtained from six measurements of every treatment from three independent experiments.

### Ethics statement

All animal procedures were performed according to the guidelines of the Canadian Council on Animal Care and approved by the Institutional Review Board of the University of British Columbia (approval #A10-0295).

### Intramuscular orthotopic xenograft murine model

Animal experiments were performed as described previously [15, 18]. Mice were subdivided into four treatment arms: RT112luc control treatment (n = 15), RT112luc dasatinib treatment (n = 15), RT112^r^GEMCI^20^luc control treatment (n = 15), and RT112^r^GEMCI^20^luc dasatinib treatment (n = 14). Six-week-old male nude mice (Harlan Laboratories, Indianapolis, IN, USA) were anesthetized with isoflurane (2 Vol. %) and analgesia was provided by subcutaneous injection with buprenorphine and meloxicam (Boehringer Ingelheim, Burlington, ON, Canada). After disinfection of the abdominal wall with chlorhexidine, a low transverse laparotomy was made and the urinary bladder was extracorporealized. 50 μl of a cell suspension containing 5 × 10^5^ cells were directly injected into the bladder wall. The incision was closed with suture. Bioluminescence was used to quantify tumor burden and was measured on the xenogen IVIS spectrum imaging system after intraperitoneal injection of 200 μg/kg luciferin (Caliper Lifesciences). Images were taken at 10 and 14 min after luciferin injection and the average counts were used for statistical analysis. Evaluation of bioluminescence in this orthotopic model showed that 10 × 10^8^ photons/s correlate to 10 mm^3^ tumor volume [19]. Bioluminescence imaging was performed on day 5 after tumor inoculation and mice were divided into equal treatment groups based on tumor burden. Treatment started the following day and imaging was repeated every 4 days. Necropsy was performed after 4 weeks. Dasatinib was administered by oral gavage with an in vivo relevant dose of 20 mg/kg body weight (BW) twice daily [20] in sodium citrate/citric acid (Sigma-Aldrich, St. Louis, MO, USA). Vehicle treatment was prepared and administered in an identical manner, but without dasatinib. All experiments were performed at least in triplicate.

### Haematoxylin and eosin staining (H&E)

All samples were fixed in buffered 4 % formalin (pH7.4) and embedded in paraffin. We used standard procedures for deparaffinization and rehydration. Slides were cut at a microtome into 3 μm slices. H&E staining were performed according to standard.

### Statistical analysis

Results are expressed as mean ± standard deviation (SD) of at least three independent experiments. For statistical analysis student´s *t* test, analysis of variance (ANOVA), and Student–Newman–Keuls-Test were performed whenever applicable. Significance was defined at values of p ≤ 0.05.

## Results

### Growth characteristics and sensitivity to gemcitabine of RT112 and RT112^r^GEMCI^20^ cells

**Fig. 1:**
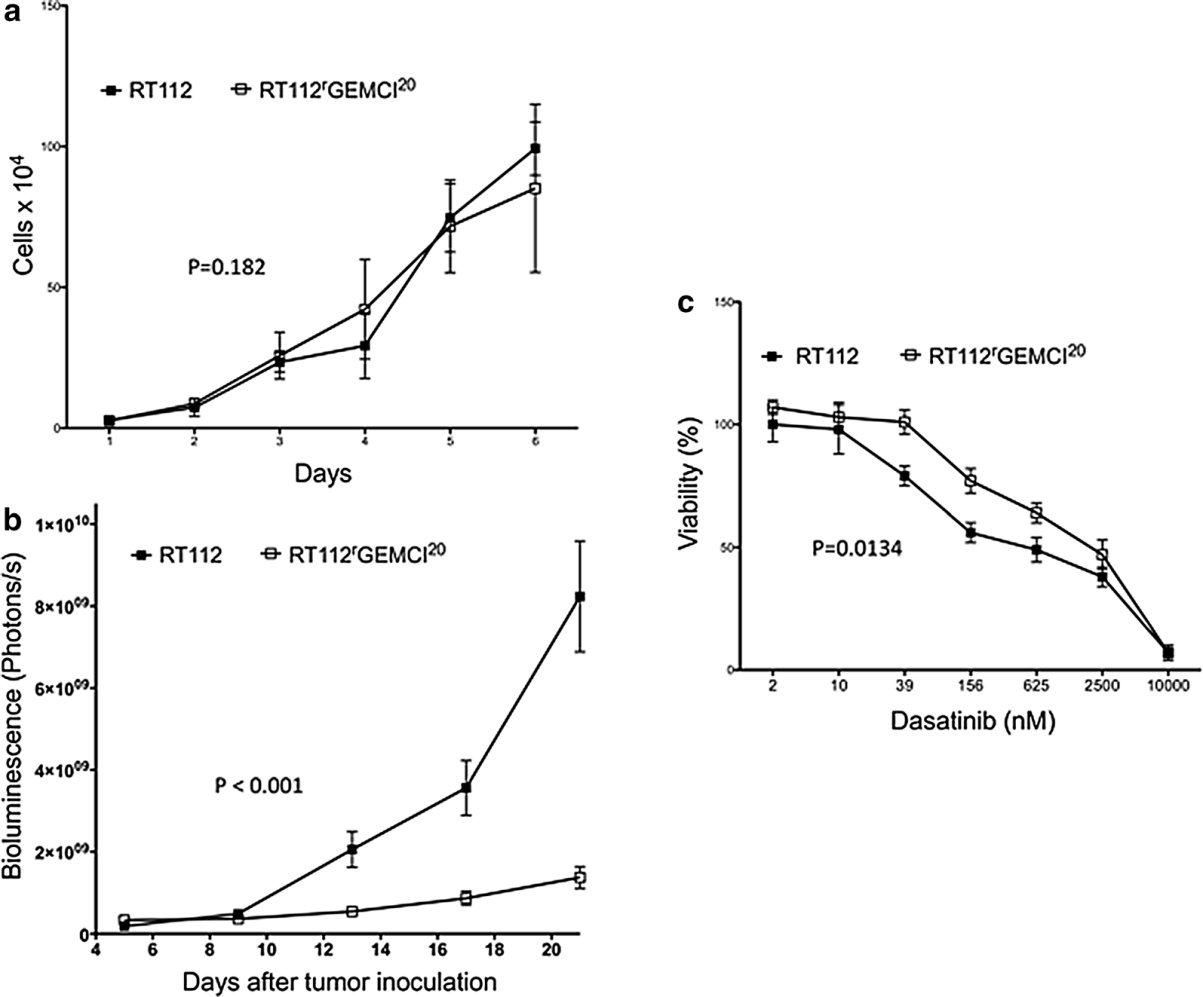
**a** Growth kinetics of the urothelial cancer cell line RT112 and its gemcitabine-resistant sub-line RT112^r^GEMCI^20^ in cell culture. On day 0, both cell lines started with 4000 cells per milliliter. **b** In vivo growth kinetics of RT112luc and RT112^r^GEMCI^20^luc tumors in an orthotopic nu/nu mouse xenograft model as determined by bioluminescence. 10 × 10^8^ photons/s correlate to 10 mm^3^ tumor volume (21). **c** Concentration-dependent effects of dasatinib on the viability of the urothelial cancer cell line RT112 and its gemcitabine-resistant sub-line RT112^r^GEMCI^20^ as indicated by MTT assay after 120 h of incubation

RT112 and RT112^r^GEMCI^20^ cells showed similar growth kinetics with doubling times of 23.28 ± 2.88 h in RT112 cells and 25.2 ± 3.12 h in RT112^r^GEMCI^20^ cells. Transduction with the luciferase plasmid, which is needed to monitor in vivo tumor growth, did not alter cell growth kinetics (doubling time RT112luc: 24.72 ± 5.04 h; doubling time RT112^r^GEMCI^20^luc: 27.36 ± 3.6 h) (Fig. 1a; Table 1). RT112^r^GEMCI^20^ cells (IC_50_ = 125.40 ± 29.78 ng/ml) displayed a 77-fold increased resistance to gemcitabine compared to parental RT112 cells (IC_50_ = 1.63 ± 0.55 ng/ml). There was a similar sensitivity to gemcitabine in the luciferase-transduced cell lines RT112luc and RT112^r^GEMCI^20^luc compared to RT112 and RT112^r^GEMCI^20^ [RT112luc (IC_50_: 1.94 ± 0.24) and RT112^r^GEMCI^20^luc (IC_50_: 114.39 ± 14.52)] (Table 1).

**Table 1:** Comparison of growth kinetics (doubling time) of RT112, RT112^r^GEMCI^20^, RT112luc, and RT112^r^GEMCI^20^luc cell lines and cell viability (IC_50_) after treatment with gemcitabine

Cell line	Doubling time (h)	IC_50_ (ng/ml)
RT112	23.28 ± 2.88	1.63 ± 0.55
RT112^r^GEMCI^20^	25.2 ± 3.12	125.40 ± 29.78
RT112luc	24.72 ± 5.04	1.94 ± 0.24
RT112^r^GEMCI^20^luc	27.36 ± 3.6	114.39 ± 14.52

### Growth characteristics of RT112luc and RT112^r^GEMCI^20^luc tumors in vivo

In vivo, growth of RT112luc and RT112^r^GEMCI^20^luc tumors was investigated in an established murine orthotopic xenograft model [15, 18, 19, 21]. RT112^r^GEMCI^20^luc cells were used for the first time in this orthotopic model. Nude mice received either 5 × 10^5^ RT112luc or RT112^r^GEMCI^20^luc cells directly injected into the bladder wall. Tumor take rates were 90 % (27 out of 30 animals) for RT112luc cells and 100 % (30 out of 30 animals) for RT112^r^GEMCI^20^luc cells. RT112^r^GEMCI^20^luc xenografts grew substantially slower than RT112luc xenografts resulting in a 6-fold smaller average tumor volume at day 25 (p < 0.0001) (Fig. 1b).

### In vitro effects of dasatinib on RT112 and RT112^r^GEMCI^20^ cells

RT112^r^GEMCI^20^ cells (IC_50_: 1081.1 ± 239.2 nM) showed a 3.1-fold decreased sensitivity to dasatinib compared to parental RT112 cells (IC_50_: 349.2 ± 67.2 nM). The concentration-dependent drug response from a representative experiment is shown in Fig. 1c.

**Fig. 2:**
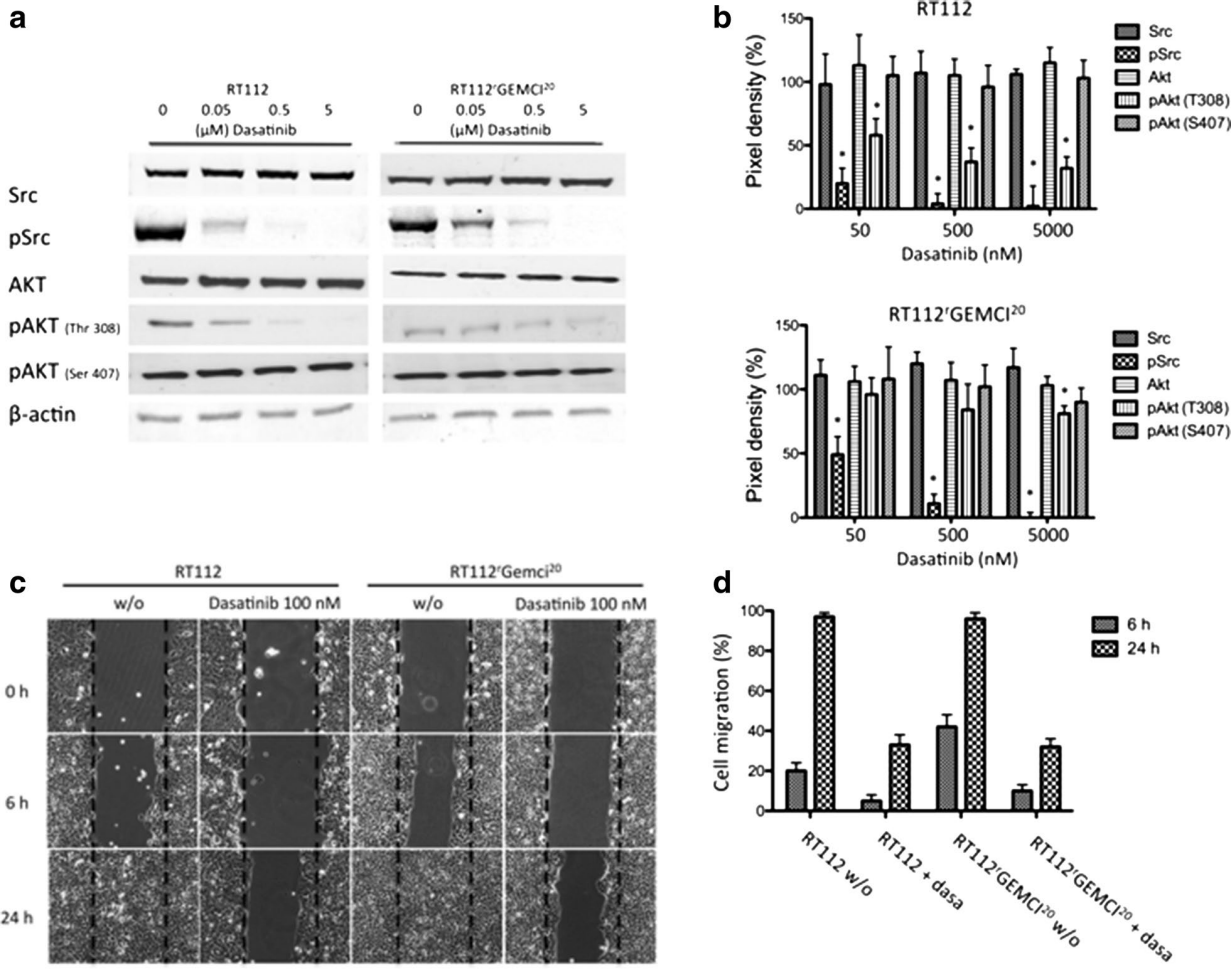
**a** Representative western blots showing cellular levels of Src, pSrc, Akt, pAkt, and β-actin in RT112 and RT112^r^GEMCI^20^ cells after 24 h incubation with dasatinib. **b** Pixel density of western blots is given in percentage compared to untreated cell line (100 %). One of three independent experiments is shown here. *p ≤ 0.05 vs untreated cell line. **c** Wound healing migration assay. Effects of dasatinib (100 nM) treatment on RT112 or RT112^r^GEMCI^20^ cells were evaluated. Images of the closure of the scratch were captured at 0, 6, and 24 h. Representative images of scratch wound assays were shown (magnification ×100). **d** Quantification of wound repair was obtained from six measurements of every treatment from three independent experiments

In cell culture, dasatinib caused in RT112 and in RT112^r^GEMCI^20^ cells a dose-dependent reduction of Src phosphorylation. Phosphorylation of Akt (Thr308) was also reduced in both cell lines with a stronger inhibition in the chemo-naive cell line. No inhibition of pAkt (Ser473) was detected (Fig. 2a, b).

Since Src inhibition was described to increase migration in urothelial cancer [12], we evaluated migratory behavior using a wound healing scratch assay in RT112 and RT112^r^GEMCI^20^ cells with and without dasatinib treatment. Results indicated a similar time-dependent migration inhibition in response to dasatinib in RT112 and RT112^r^GEMCI^20^ cells resulting in >60 % inhibition of migration in both cell lines relative to untreated control (p < 0.0001) (Fig. 2c, d).

### Effects of dasatinib treatment in RT112luc and RT112^r^GEMCI^20^luc xenografts

**Fig. 3:**
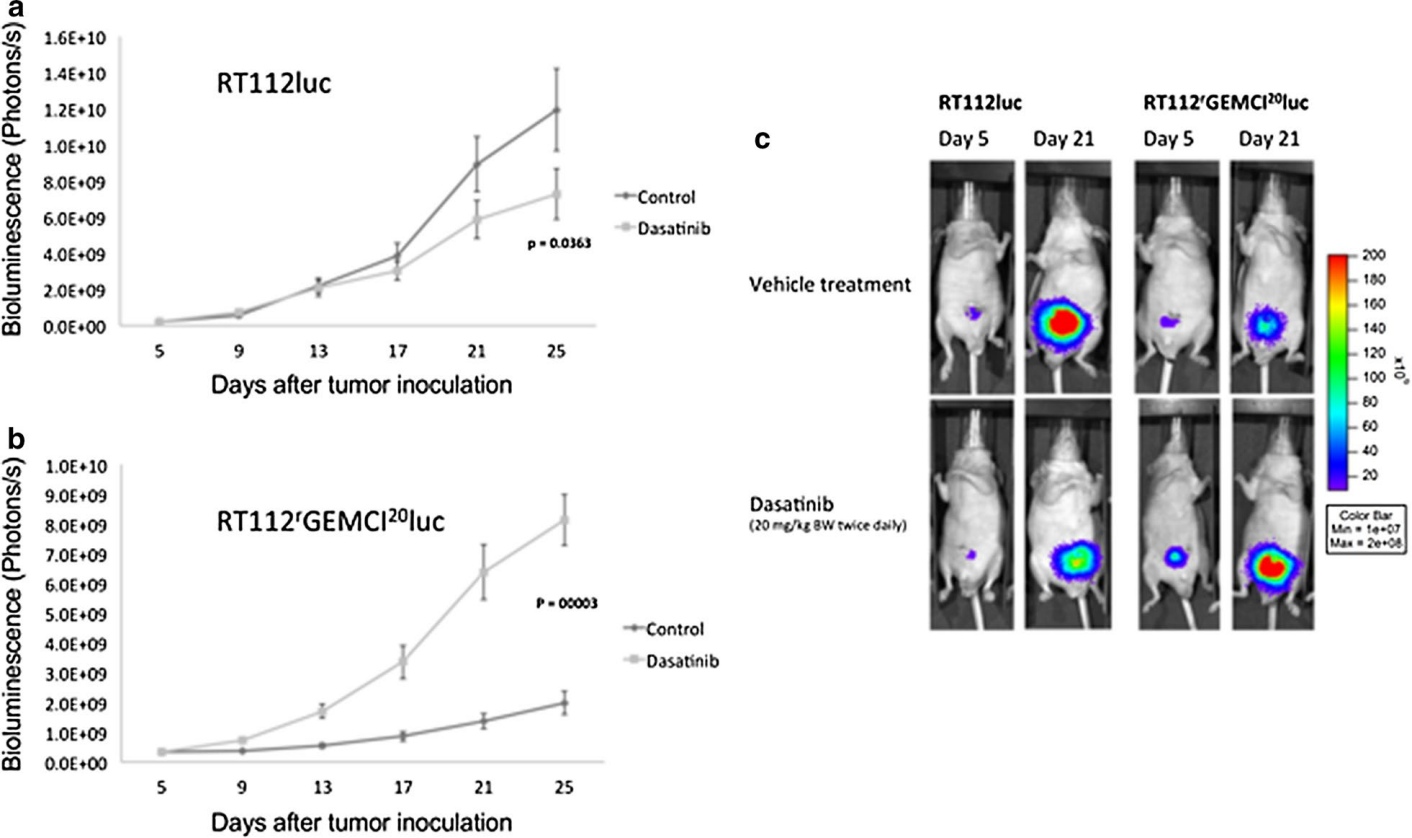
Tumor growth kinetics of murine orthotopic **a** RT112luc and **b** RT112^r^GEMCI^20^luc xenografts. Dasatinib was given per oral gavage at a dose of 20 mg/kg body weight (BW) twice daily. Control animals were vehicle-treated in the same way. 10 × 10^8^ photons/s correlate to 10 mm^3^ tumor volume (21). **c** Representative photographs of treated and untreated RT112luc and RT112^r^GEMCI^20^luc xenografts with fluorescent bladder tumors

Dasatinib treatment using 20 mg/kg twice daily [20] caused a reduction of RT112luc xenograft size by 39 % (p = 0.036) relative to untreated control at day 25. In contrast, dasatinib treatment induced a 4-fold increase in RT112^r^GEMCI^20^luc tumor size compared to vehicle treated RT112^r^GEMCI^20^luc xenografts at day 25 (p < 0.001) (Fig. 3).

**Fig. 4:**
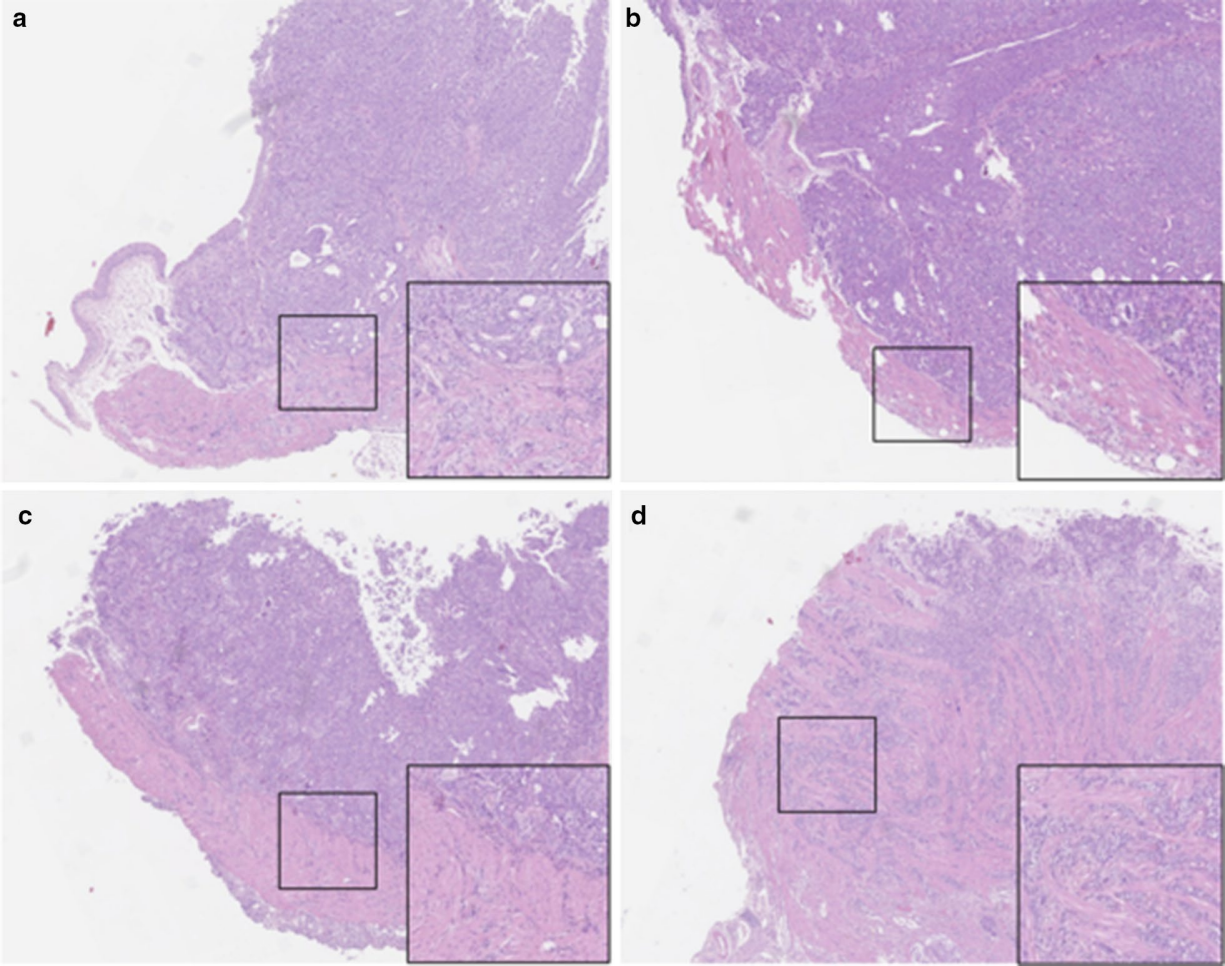
H&E staining of four representative tumors (magnification ×5). **a** RT112luc xenograft from a vehicle-treated mouse. **b** RT112luc xenograft from a dasatinib (20 mg/kg BW twice daily)-treated mouse. **c** RT112^r^GEMCI^20^luc xenograft from a vehicle-treated mouse. **d** RT112^r^GEMCI^20^luc xenograft from a dasatinib (20 mg/kg BW twice daily)-treated mouse

Tumors were sampled and H&E staining was performed at day 25. Untreated RT112luc tumors displayed muscle-invasive growth (pT2) (Fig. 4a). In contrast, no signs of muscle-invasive growth (pT1) were detected in dasatinib-treated RT112luc tumors (Fig. 4b). The opposite pattern was observed in RT112^r^GEMCI^20^luc tumors. Untreated RT112^r^GEMCI^20^luc tumors did not show muscle-invasive growth (pT1) (Fig. 4c), while dasatinib-treated RT112^r^GEMCI^20^luc xenografts displayed muscle invasive growth (pT2) (p < 0.001). In dasatinib-treated RT112^r^GEMCI^20^luc xenografts, the bladder muscle was completely infiltrated by the tumor, and the bladder wall was not clearly visible anymore (Fig. 4d).

## Discussion

In this study, we compared the effects of the Src inhibitor dasatinib on the urothelial cancer cell line RT112 and its gemcitabine-resistant sub-line RT112^r^GEMCI^20^ in cell culture and in an orthotopic bladder cancer xenograft model in mice.

In cell culture, both cell lines displayed similar growth kinetics. Dasatinib inhibited Src phosphorylation in RT112 and RT112^r^GEMCI^20^ cells at low nanomolar concentrations similar to those that had already been described to affect Src phosphorylation [9]. While dasatinib had previously been shown to interfere with the phosphorylation of Akt (Thr308 and Ser473) in squamous cell lung cancer [22], we only detected inhibition of phosphorylation of Akt (Thr308). The reasons for this may be the consequence of cell type-specific differences between the investigated models. Although dasatinib exerted similar effects on Src signaling in RT112 and RT112^r^GEMCI^20^ cells, its effects on cell viability differed between the two cell lines. The effective concentrations of dasatinib in RT112 cells (IC_50_ of 349.2 ± 67.2 nM) were in the range of those previously described for the treatment of urothelial cancer cells [9]. However, in RT112^r^GEMCI^20^ cells the IC_50_ value was about 3-fold higher (1081.1 ± 239.2 nM). This suggests that RT112^r^GEMCI^20^ cells have acquired resistance mechanisms that interfere with antitumoral effects of dasatinib downstream of Src signaling. In accordance, dasatinib treatment resulted in reduced inhibition of the phosphorylation of the Src downstream kinase Akt (Thr 308) in RT112^r^GEMCI^20^ cells compared to RT112 cells.

In nude mice, RT112^r^GEMCI^20^luc cells formed about 6-fold smaller tumors than RT112luc cells. The reasons for this remain unclear. However, it is known that different cell lines may differ in their interaction with the tissue environment in animal models resulting in discrepancies in the growth kinetics [23, 24].

The most striking differences were found after oral dasatinib treatment of mice bearing orthotopic RT112luc or RT112^r^GEMCI^20^luc xenografts. Dasatinib treatment of RT112luc bladder tumors resulted in a significant reduction of tumor size relative to untreated control. In contrast, dasatinib treatment of RT112^r^GEMCI^20^luc bladder tumors resulted in a dramatic increase of tumor growth. In addition, the dasatinib treatment prevented muscle-invasive growth of RT112luc xenografts, but strongly induced muscle-invasive growth of RT112^r^GEMCI^20^luc xenografts. The mechanistic reasons underlying this discrepancy remain elusive. Wu et al. [11] had reported that loss of Src increases metastasis formation in a bladder cancer model. Similar to this report, Thomas et al. [12] showed that Src enhances urothelial cancer cell migration and metastasis formation. However, dasatinib inhibited RT112 and RT112^r^GEMCI^20^ cell migration in a similar manner, similarly to other studies that reported on the effects of Src inhibition on cancer cell migration in models from different cancer entities [25–28]. Therefore, it seems unlikely that the dasatinib-induced increased invasive growth of RT112^r^GEMCI^20^luc cells in the xenograft model may be caused by dasatinib-induced enhanced RT112^r^GEMCI^20^luc cell migration.

## Conclusions

We present the first study that investigated the effects of dasatinib on urothelial cancer cells with acquired resistance to gemcitabine. In cell culture, gemcitabine-resistant RT112 cells were less sensitive to dasatinib than parental RT112 cells. Notably, parental RT112 cells and gemcitabine-resistant RT112 cells displayed an unexpected opposite response to dasatinib in an orthotopic xenograft model in mice. While dasatinib inhibited tumor growth and muscle invasion by parental RT112 cells, it increased tumor growth and muscle invasion by gemcitabine-resistant RT112 cells. Thus, our data do not generally support the use of dasatinib for the treatment of urothelial cancer, in particular not for therapy-refractory cases after first-line chemotherapy with gemcitabine. However, further studies will need to show whether similar effects are obtained in additional models of (acquired gemcitabine resistance in) urothelial cancer. If such studies suggested that some urothelial cancer diseases may benefit from dasatinib therapy, biomarkers would need to be identified that enable the prediction of the response of individual urothelial cancer diseases to dasatinib.

## Abbreviations

MTT: 3-(4,5-dimethylthiazol-2-yl)-2,5-diphenyltetrazolium bromide
IC_50_: half maximal inhibitory concentration
GC: gemcitabine and cisplatin
DT: doubling time
H&E: haematoxylin and eosin staining
SD: standard deviation
ANOVA: analysis of variance
